# Hyperparasitoids exploit herbivore-induced plant volatiles during host location to assess host quality and non-host identity

**DOI:** 10.1007/s00442-019-04352-w

**Published:** 2019-02-05

**Authors:** Antonino Cusumano, Jeffrey A. Harvey, Marcel Dicke, Erik H. Poelman

**Affiliations:** 1 Laboratory of Entomology, Department of Plant Sciences, Wageningen University, Droevendaalsesteeg 1, 6708 PB Wageningen, The Netherlands; 2 Department of Terrestrial Ecology, Netherlands Institute of Ecology (NIOO-KNAW), Droevendaalsesteeg 1, 6708 PB Wageningen, The Netherlands; 3 Section Animal Ecology, Department of Ecological Sciences, VU University Amsterdam, De Boelelaan 1085, 1081 HV Amsterdam, The Netherlands

**Keywords:** Hyperparasitoid foraging behavior, Non-host parasitoid species, Fourth trophic level organisms, Multitrophic interactions, Plant-based food web

## Abstract

Although consumers often rely on chemical information to optimize their foraging strategies, it is poorly understood how top carnivores above the third trophic level find resources in heterogeneous environments. Hyperparasitoids are a common group of organisms in the fourth trophic level that lay their eggs in or on the body of other parasitoid hosts. Such top carnivores use herbivore-induced plant volatiles (HIPVs) to find caterpillars containing parasitoid host larvae. Hyperparasitoids forage in complex environments where hosts of different quality may be present alongside non-host parasitoid species, each of which can develop in multiple herbivore species. Because both the identity of the herbivore species and its parasitization status can affect the composition of HIPV emission, hyperparasitoids encounter considerable variation in HIPVs during host location. Here, we combined laboratory and field experiments to investigate the role of HIPVs in host selection of hyperparasitoids that search for hosts in a multi-parasitoid multi-herbivore context. In a wild *Brassica oleracea*-based food web, the hyperparasitoid *Lysibia nana* preferred HIPVs emitted in response to caterpillars parasitized by the gregarious host *Cotesia glomerata* over the non-host *Hyposoter ebeninus*. However, no plant-mediated discrimination occurred between the solitary host *C. rubecula* and the non-host *H. ebeninus*. Under both laboratory and field conditions, hyperparasitoid responses were not affected by the herbivore species (*Pieris brassicae* or *P. rapae*) in which the three primary parasitoid species developed. Our study shows that HIPVs are an important source of information within multitrophic interaction networks allowing hyperparasitoids to find their preferred hosts in heterogeneous environments.

**Electronic supplementary material:**

The online version of this article (10.1007/s00442-019-04352-w) contains supplementary material, which is available to authorized users.

## Introduction

Consumers often forage in heterogeneous environments in which resources of different quality are interspersed among other resources that are nutritionally unsuitable. The efficiency in finding and exploiting nutritionally suitable resources is crucial for maximizing the consumers’ fitness (Charnov 1976; Pyke 1984). The problem of foraging in heterogeneous environments is widespread among consumers structured within food webs; for example, herbivorous and carnivorous insects that are part of plant-based food webs have to find resources which are commonly embedded within larger patches of non-resources (Aartsma et al. 2017, 2019). Herbivores need to find their food plants among a diverse array of non-food plants, whereas carnivores such as parasitoids have to find their herbivore hosts and discriminate between non-infested and infested plants (Bruce and Pickett 2011; De Rijk et al. 2013). To orient towards suitable resources in structurally complex vegetation, herbivorous and carnivorous insects often rely on chemical sources of information among which plant volatiles play a key role (Bruce et al. 2005; Clavijo McCormick et al. 2012; Webster and Cardé 2017). Herbivores have been shown to exploit specific ratios of ubiquitous plant volatiles to locate host–plant species (Bruce et al. 2005; Webster et al. 2010) whereas parasitoids use plant volatiles induced by herbivore attack (HIPVs) as cues for host location (Mumm and Dicke 2010; Clavijo McCormick et al. 2012; Turlings and Erb 2018).

Plant-based food webs usually go beyond the third trophic level (Bukovinszky et al. 2008; Harvey et al. 2009; Frago 2016; Sanders et al. 2016; Seibold et al. 2018). Obligate hyperparasitoids are a common group of insects in the fourth trophic level which lay their eggs in or on the body of other parasitoid hosts. Primary hyperparasitoids develop on parasitoid host larvae whereas secondary hyperparasitoids attack parasitoid prepupae or pupae (Sullivan 1987; Sullivan and Volkl 1999). The foraging behavior of hyperparasitoids has received little attention compared to insects in lower trophic levels, such as herbivores and primary parasitoids. Despite this paucity of information, hyperparasitoids clearly must also deal with several constraints when foraging. For one thing, their primary parasitoid hosts are more scarce (and thus presumably harder to find) than herbivore hosts of primary parasitoids. Moreover, parasitoid host larvae do not feed on plants and, therefore, they are inconspicuous and often concealed within the body of their herbivore hosts (Sullivan and Volkl 1999; Brodeur 2000). Additional challenges faced by hyperparasitoids include the fact that the same herbivore species may be attacked by several parasitoid species which differ in host quality and some of them may not even be suitable for hyperparasitoid offspring development (Harvey 2005). Furthermore, the same parasitoid host may develop on/in different herbivore species. Because of all these challenges, hyperparasitoids clearly need to make the best use of all available information when searching for hosts to optimize their foraging efficiency.

Previous studies show that the hyperparasitoid *Lysibia nana* can use plant volatiles emitted in response to feeding by parasitized caterpillars to locate their parasitoid hosts and discriminate HIPVs according to the parasitization status of the caterpillar feeding on the plant (Poelman et al. 2012; Zhu et al. 2015). The changes in HIPV composition that allow hyperparasitoids to find their hosts are mainly driven by an alteration in the composition of oral secretions of caterpillar hosts as a result of being parasitized (Poelman et al. 2011; Shikano et al. 2017; Tan et al. 2018; Zhu et al. 2018), which in turn play a key role in herbivore recognition and plant defense signaling (Bonaventure et al. 2011; Bonaventure 2012; Rivera-Vega et al. 2017). Interestingly, the specificity of the parasitoid signature is reflected in the physiology of the caterpillar in such a way that each parasitoid species induces a specific effect on herbivore oral secretions and plant responses to herbivory (Poelman et al. 2011; Zhu et al. 2015; Kaplan et al. 2016; Ode et al. 2016). As a consequence, HIPVs may convey valuable information that hyperparasitoids can use to assess the identity of the parasitoid host and, possibly, even parasitoid host quality and suitability (Poelman and Kos 2016). Yet, how hyperparasitoids forage in complex environments containing hosts and non-hosts has not been explored.

**Fig. 1:**
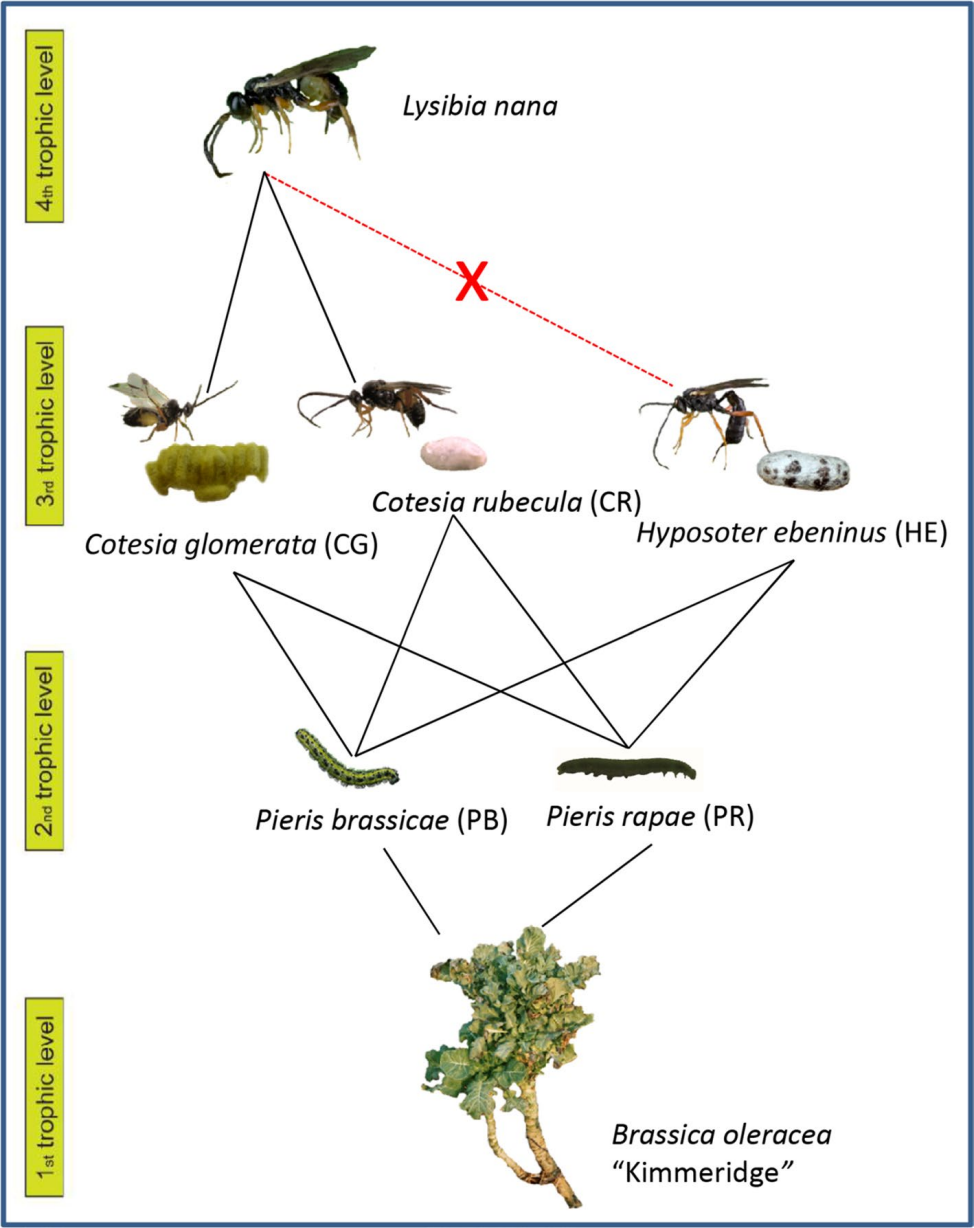
Overview of the four-trophic-level food web used in this study. The hyperparasitoid *Lysibia nana* attacks cocoons of *Cotesia glomerata* (CG) and *C. rubecula* (CR) but it cannot develop in *Hyposoter ebeninus* (HE) which represents a non-host species for the hyperparasitoid. In turn, each primary parasitoid species can develop in both *Pieris brassicae* (PB) and *P. rape* (PR) caterpillars which feed on the wild *Brassica oleracea* “Kimmeridge” population

In this study, we used a food web based on wild *Brassica oleracea* to investigate the foraging behavior of hyperparasitoids in a scenario in which hosts of different quality and non-host parasitoid species develop in different herbivore species feeding on neighboring plants (Fig. 1). As focal hyperparasitoid species we used *L. nana* which is a specialist attacking pupae of parasitoids in the genus *Cotesia*. In cabbage fields of The Netherlands, *L. nana* mainly attacks the gregarious parasitoid *Cotesia glomerata* and the solitary parasitoid *C. rubecula*; both parasitoid species parasitize the co-occurring herbivorous caterpillars *Pieris brassicae* and *P. rapae* (Geervliet et al. 1998, 2000). However, the solitary parasitoid *Hyposoter ebeninus* can also attack both *P. brassicae* and *P. rapae* and is locally sympatric with *C. glomerata* and *C. rubecula* (Feltwell 1982; Poelman et al. 2014). As *H. ebeninus* is not a suitable resource for *L. nana* offspring development, it can potentially disrupt HIPV exploitation and limit the hyperparasitoid’s foraging efficiency if no discrimination between hosts and non-host parasitoids is displayed. Furthermore, the two *Cotesia* species represent hosts of different quality for *L. nana* in terms of maternal fitness investments. In fact, the gregarious *C. glomerata* is a high-quality resource because a *L. nana* female will often parasitize most of the gregarious brood in sequence during a single foraging bout. By contrast, the solitary *C. rubecula* represents a host of lower quality as it allows only a single reproductive opportunity with the consequence that an *L. nana* female has to disperse after attacking a parasitized caterpillar.

Plant-mediated discrimination between hosts of different quality and non-host parasitoid species may also be affected by the herbivore in which parasitoid larvae are developing: because the two *Pieris* species display different feeding behavior (*P. brassicae* caterpillars feed gregariously on the plants whereas *P. rapae* caterpillars feed individually), the way the parasitized herbivore species interact with the plant may differ with consequences for variation in HIPV blends and hyperparasitoid foraging behavior (Poelman et al. 2012).

As previous studies have shown that *L. nana* exploits HIPVs during host location (Poelman et al. 2012; Zhu et al. 2015), here we tested the hypothesis that HIPVs allow hyperparasitoids to orient themselves in complex environments by conveying information about the identity of the parasitoid species developing in different herbivore species. We combined laboratory and field investigations to specifically address the following questions: (1) whether *L. nana* hyperparasitoids discriminate between hosts (*C. glomerata*, *C. rubecula*) and non-hosts (*H. ebeninus*) based on HIPVs emitted in response to feeding by parasitized caterpillars; (2) whether the herbivore species (*P. brassicae, P. rapae*) in which hosts and non-host parasitoid larvae develop induce variation in HIPVs that affect the foraging behavior of *L. nana* hyperparasitoids.

## Materials and methods

### Plants and insects

Seeds of the wild *Brassica oleracea* population ‘Kimmeridge’ (Dorset, UK, 50°360N, 2°070W) were grown in a glasshouse compartment (22 ± 3 °C, 50–70% relative humidity and 16:8 h L:D photoperiod). In the field, the Kimmeridge population is attacked by different herbivores and colonization by *Pieris brassicae* and *P. rapae* is frequent (Newton et al. 2010). The Kimmeridge population was selected for this study due to its strong induced responses to *Pieris* herbivory compared to other *B. oleracea* populations (Gols et al. 2008).

The herbivores (*P. brassicae* and *P. rapae*) and parasitoids (*C. glomerata* and *C. rubecula*) were originally collected from field sites near Wageningen University, The Netherlands whereas the colony of *H. ebeninus* was originally collected as cocoons from cabbage fields near the University of Rennes, France (Harvey et al. 2010). We confirmed the non-host status of *H. ebeninus* by offering *H. ebeninus* cocoons to *L. nana* females. We observed that the hyperparasitoids made only occasional visits to these cocoons and never attempted to oviposit in the cocoons. None of the *H. ebeninus* cocoons exposed for several days in a cage with *L. nana* yielded a new generation of *L. nana* emerging from the cocoons. Unparasitized *Pieris* species were reared in glasshouse compartments (22 ± 1 °C, 50–70% relative humidity and 16:8 h L:D photoperiod) on cabbage plants (*B. oleracea* var *gemmifera* cv. Cyrus). To prepare parasitized caterpillars for the induction treatments, individual first instar *P. brassicae* or *P. rapae* caterpillars were exposed to a single female parasitoid (*C. glomerata*, *C. rubecula* or *H. ebeninus*) which was allowed to parasitize the caterpillars in a glass vial. The *Pieris* caterpillar was considered to be parasitized when the wasp had inserted her ovipositor in the herbivore for at least 5 s in the case of the gregarious *C. glomerata* (which lays about 15–40 eggs per caterpillar) or for 1 s in the case of the solitary *C. rubecula* and *H. ebeninus* (Poelman et al. 2011, 2014). No more than ten caterpillars were offered to a single female parasitoid to avoid possible negative effects caused by depletion of the parasitoid’s egg load. Parasitized caterpillars were reared on cabbage plants until used for induction treatments in laboratory and field experiments.

The hyperparasitoid *Lysibia nana* was originally recovered from *C. glomerata* cocoons collected from field sites near Wageningen University, The Netherlands, and was reared on *C. glomerata* cocoons in the absence of plant- and herbivore-derived cues. When possible, all insect colonies were annually refreshed with field collected material.

### Y-tube olfactometer bioassays

Wild *B. oleracea* plants used for the olfactometer bioassays were 5-weeks-old and treated for 24 h as described by Poelman et al. (2012) before the tests were carried out. Briefly, plants were left undamaged (UD) or infested with either two unparasitized fourth instar *P. brassicae* (PB) or *P. rapae* (PR) caterpillars or two-fourth instar *Pieris* caterpillars that contained fully grown parasitoid larvae of either *C. glomerata* (CG), *C. rubecula* (CR) or *H. ebeninus* (HE) as a result of parasitism of the caterpillar in their first instar. We carried out two parallel groups of olfactometer bioassays according to herbivore identity (i.e., all pairwise combinations using plants induced only with *P. brassicae* or with *P. rapae* caterpillars, respectively). The same replicates per experimental day were tested with the different herbivore species.

In a first set of bioassays, we investigated *L. nana* preferences for plant odors emitted in response to damage by unparasitized caterpillars (PB or PR) or caterpillars parasitized by non-host parasitoids (PB-HE or PR-HE) vs. odor of undamaged control plants (UD). In a second set of bioassays, we investigated whether hyperparasitoids discriminate between plant volatiles induced by caterpillars carrying parasitoid hosts (PB-CG, PB-CR or PR-CG, PR-CR) or non-hosts (PB-HE or PR-HE) over plants damaged by unparasitized caterpillars (PB or PR). In a third set of bioassays, we used only plants damaged by parasitized herbivores to investigate whether *L. nana* females discriminate the identity of the parasitoid species growing inside the caterpillars based on HIPV composition. Each treatment combination was replicated with seven plant pairs and ten hyperparasitoids per plant pair (*n* = 70 hyperparasitoid females per treatment).

Shortly before *L. nana* females were tested for their behavioral response to plant volatiles in Y-tube olfactometer bioassays, we removed caterpillars and their feces from the plants and placed the plants in one of two glass jars (30 l each) that were connected to the two olfactometer arms. A charcoal-filtered airflow (4 l/min) was led through each arm of the Y-tube olfactometer, and a single wasp was released at the base of the stem Section (3.5 cm diameter, 22 cm length) in each test. The Y-tube olfactometer setup was illuminated with four fluorescent tubes (FTD 32 W/84 HF, Pope, The Netherlands). Wasps that passed a set line at the end of one of the olfactometer arms within 10 min and stayed there for at least 15 s were considered to have made a choice. To compensate for unforeseen asymmetry in the setup, we swapped the jars containing the plants after testing five wasps and replaced the set of plants by a new set of plants after testing ten wasps. Each wasp was only used once.

### Common garden experiment

Four-week-old plants were transplanted into the field with 1 × 1 m spacing between plants and allowed to adjust to field conditions for 1 week. Thereafter, the plants were subjected to one of the nine following induction treatments: (1) not treated with herbivory (i.e., undamaged controls, UD); (2) infested individually with either two unparasitized first instar *P. brassicae* (PB) or (3) *P. rapae* caterpillars (PR); (4) two *C. glomerata*–parasitized *P. brassicae* (PB-CG) or (5) *P. rapae* caterpillars (PR-CG); (6) two *C. rubecula*–parasitized *P. brassicae* (PB-CR) or (7) *P. rapae* caterpillars (PR-CR); (8) two *H. ebeninus*–parasitized *P. brassicae* (PB-HE) or (9) *P. rapae* caterpillars (PR-HE). Unparasitized and parasitized caterpillars were allowed to feed on plants for 10 days, which was approximately the whole development period of the koinobiont endoparasitoid larvae used in this study. Each plant was covered with a fine-mesh net to avoid other herbivore infestations on the plant and to prevent the herbivores used for induction to wander off the plant.

To test the effects of plant induction by different types of herbivory on hyperparasitism, we attached *C. glomerata* cocoon clutches each consisting of about 20–30 cocoons onto the plants in the field (Poelman et al. 2012). Individual cocoon clutches of *C. glomerata* were first attached to a piece of cardboard (3 × 3 cm) with a small droplet of glue (HEMA, The Netherlands). We removed nets and caterpillars just before attaching the cardboards carrying the parasitoid pupae with a pin. We attached five cocoon clutches onto each plant. To increase the abundance of *L. nana* in the field, 100 laboratory-reared females were released at the four cardinal points 3 m away from the edges of the experimental field immediately after the cocoon clutches were attached to the plants. Cocoon clutches were recollected after 5 days of exposure to the hyperparasitoid communities in the field. Subsequently, they were individually kept in the laboratory in 2-mL Eppendorf vials that were closed with cotton wool. The Eppendorf vials were checked daily for emergence of *C. glomerata* parasitoids and hyperparasitoids. The large majority of the hyperparasitoids were identified to the species level.

A completely randomized design was applied to the field assays. We repeated the experiment five times from May until October 2016, each replicate including 100 plants that included 10 replicates of each treatment (except the UD treatment which had 20 replicates).

### Statistical analyses

Hyperparasitoid preferences for herbivore-induced plant volatiles, as tested in two-choice Y-tube olfactometer assays, were analyzed with a Generalized Linear Model (GLM) with a binomial distribution and a logit link function. When overdispersion in the model was detected, a quasi-binomial distribution was fitted. To determine whether there was a significant preference for one of the offered plants within the pairwise combination, we tested *H*0: logit = 0.

Hyperparasitoid preferences for plant volatiles induced by unparasitized and parasitized caterpillars under field conditions were analyzed using two additional GLMs. In the first model, we tested the caterpillar induction treatment as a factor with 9 levels (UD, PR, PB, PR-CG, PB-CG, PR-CR, PB-CR, PR-HE, PB-HE) including in the GLM also the effect of the replicate (five replicates). In the second model, we included the factors herbivore species (*P. rapae* or *P. brassicae*), parasitism effect (*C. glomerata*, *C. rubecula*, *H. ebeninus*, none) and replicate (five replicates) to evaluate the overall effect of parasitism and herbivore identity on hyperparasitism rates. We tested both models, using as response variables the hyperparasitism rates achieved by all species (total hyperparasitism) or by *L. nana* only. For both GLMs, we analyzed the effects at plant level, by modeling the response variable as a binomial occurrence of hyperparasitism per plant and scored presence of hyperparasitoids in cocoon clutches as “1” and absence as “0”. Additionally, to test the effects at cocoon clutch level, we modeled the response variable as the number of clutches yielding hyperparasitoids out of the fixed totals of five cocoon clutches attached to the plant. Analyses at the plant level provide insight into whether a given treatment is visited more frequently by hyperparasitoids (i.e., attraction preference). Analyses at the cocoon clutch level may provide additional indication of the number of hyperparasitoids that visited an individual plant, or whether in some treatments hyperparasitoids stay longer on the plant to parasitize multiple cocoons (i.e., arrestment preference). Data were analyzed with R statistical software (R Development Core Team 2013).

## Results

### Y-tube olfactometer bioassays

**Fig. 2:**
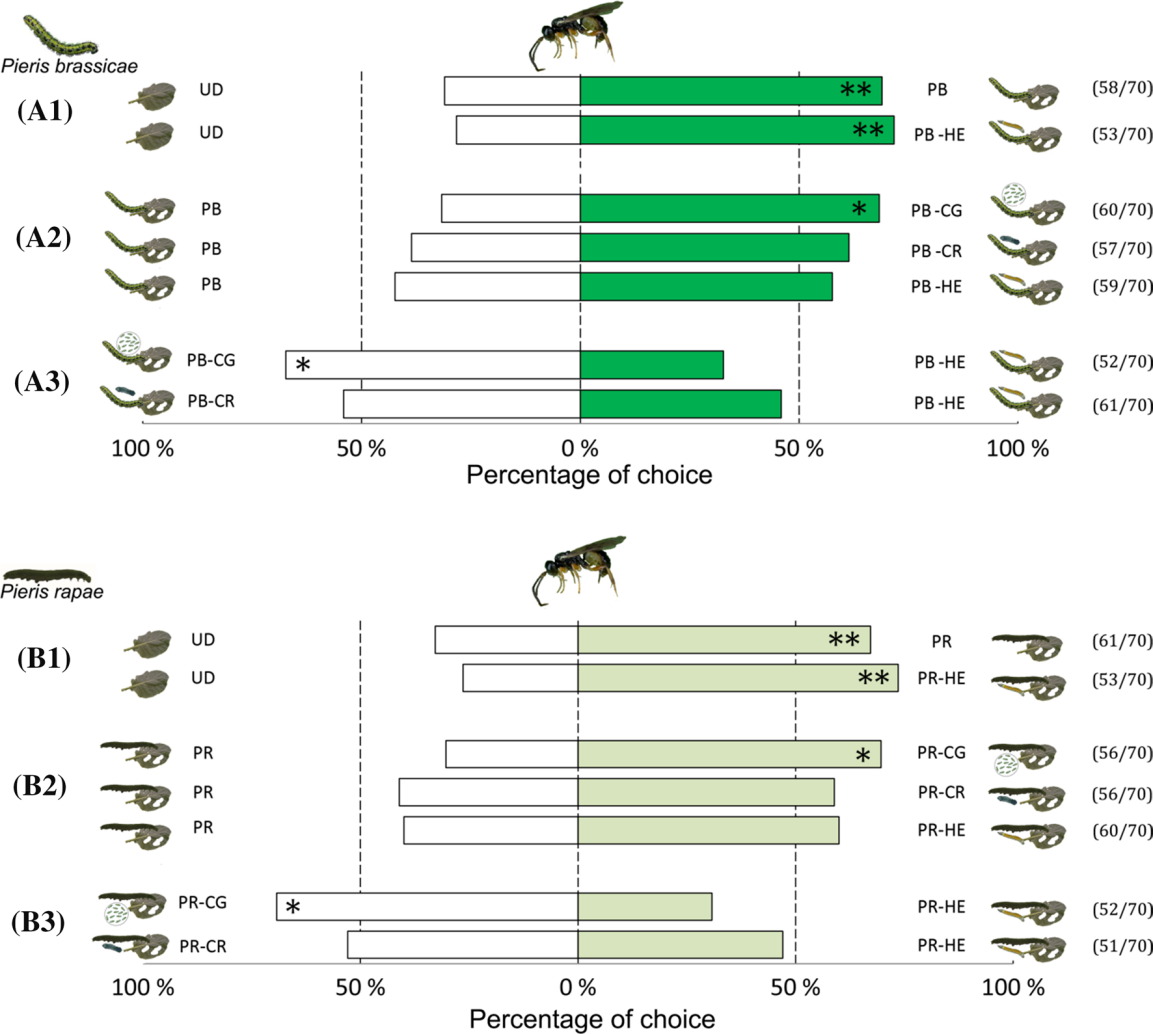
Preference of *Lysibia nana* females for herbivore-induced plant volatiles (HIPVs) in two-choice olfactometer tests. Above: olfactometer tests using *Pieris brassicae* as herbivore species comparing undamaged control plants (UD), *P. brassicae*-damaged plants (PB), plants damaged by *Hyposoter ebeninus*-parasitized *P*. *brassicae* caterpillars (PB-HE), plants damaged by *Cotesia glomerata*-parasitized *P. brassicae* caterpillars (PB-CG), plants damaged by *Cotesia rubecula*-parasitized *P. brassicae* caterpillars (PB-CR). Below: olfactometer tests using *Pieris rapae* as herbivore species comparing undamaged control plants (UD), *P. rapae*-damaged plants (PR), plants damaged by *Hyposoter ebeninus*-parasitized *P*. *rapae* caterpillars (PR-HE), plants damaged by *Cotesia glomerata*-parasitized *P. rapae* caterpillars (PR-CG), plants damaged by *Cotesia rubecula*-parasitized *P. rapae* caterpillars (PR-CR). Asterisks indicate a preference which is significantly different from a 50:50 distribution within a choice test (GLM, *P < 0.05, **P < 0.01). Numbers between brackets indicate the number of responding wasps vs. the total number of wasps tested

Hyperparasitoids preferred plant volatiles induced by unparasitized *Pieris* caterpillars over undamaged control plants (GLM, PB: *χ*^2^ = 8.34, *n* = 7, *P *= 0.0039; PR: *χ*^2^ = 7.23, *n* = 7, *P *= 0.0072) (Fig. 2a1, b1). Similarly, *L. nana* females were attracted to HIPVs emitted in response to attack by both *P. brassicae* and *P. rapae* caterpillars carrying non-host parasitoid larvae when tested against undamaged control plants (PB: *χ*^2^ = 9.98, *n* = 7, *P *= 0.0016; PR: *χ*^2^ = 11.79, *n* = 7, *P *= 0.0006).

When hyperparasitoids were offered plant odors induced by parasitized caterpillars over unparasitized caterpillars, discrimination based on the parasitism status of the attacking herbivore only occurred in the case of *C. glomerata*, regardless of the *Pieris* species (PB: *χ*^2^ = 8.34, *n* = 7, *P *= 0.0045; PR: *χ*^2^ = 8.64, *n* = 7, *P *= 0.0033) (Fig. 2a2, b2). In fact, hyperparasitoids did not discriminate between HIPVs induced by unparasitized *Pieris* caterpillars vs. HIPVs induced by caterpillars parasitized by the host *C. rubecula* (PB: *χ*^2^ = 2.96, *n* = 7, *P *= 0.0851; PR: *χ*^2^ = 1.79, *n* = 7, *P *= 0.1814) or by the non-host *H. ebeninus* (PB: *χ*^2^ = 1.37, *n* = 7, *P *= 0.2413; PR: *χ*^2^ = 2.40, *n* = 7, *P *= 0.1213).

The hyperparasitoids preferred HIPVs emitted upon herbivory by *Pieris* caterpillars parasitized by the gregarious host *C. glomerata* over the non-host *H. ebeninus* (PB: *χ*^2^ = 6.23, *n* = 7, *P *= 0.0125; PR: *χ*^2^ = 7.69, *n* = 7, *P *= 0.0055) (Fig. 2a3, b3). However, *L. nana* did not discriminate between HIPVs induced by caterpillars carrying the solitary host *C. rubecula* or the solitary non-host *H. ebeninus* (PB: *χ*^2^ = 0.41, *n* = 7, *P *= 0.5221; PR: *χ*^2^ = 0.17, *n* = 7, *P *= 0.6744).

### Common garden experiments

In our field experiment, we recovered a total of five hyperparasitoid species from *C. glomerata* cocoons among which *L. nana* was by far the most abundant (recorded in 89.1% of the cases) as shown in the Online Resource 1 of the Electronic Supplementary Material (ESM). The plant induction treatment significantly affected total hyperparasitism rates both at the plant level (Table 1) and at the cocoon clutch level (Online Resource 2, ESM).

**Table 1:** The effect of plant induction treatment on the overall hyperparasitism rates achieved on *Cotesia glomerata* cocoons at the plant level

Model factor	Deviance	Degrees of freedom	*P* value
Overall	494.81	499	
Factor			
Induction treatment (1)	37.889	8	< 0.001
Replicate (2)	48.207	4	< 0.001
(1) × (2)	16.863	32	0.9871

Hyperparasitism was modeled as a binomial occurrence of hyperparasitoids (presence = 1, absence = 0) emerging from the five cocoon clutches attached per plant

**Fig. 3:**
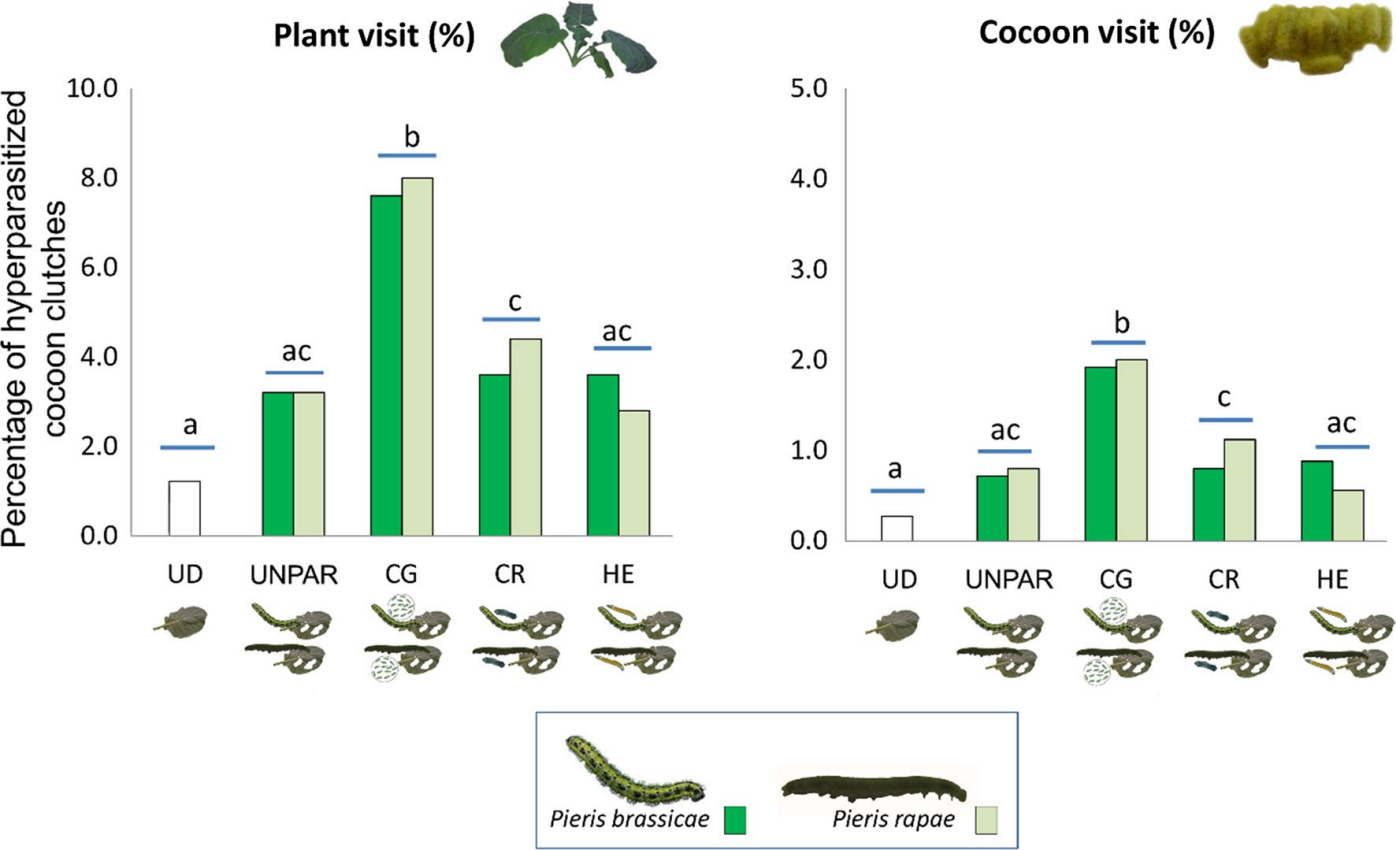
Percentage of *Cotesia glomerata* cocoon clutches that contained hyperparasitoids in the field trials either at the plant level (i.e., at least one cocoon clutch out of the five clutches attached to the plant yielded hyperparasitoids) (left) or at the individual clutch level (right). The cocoons were collected from plants that were either left untreated (UD), infested with unparasitized *Pieris* caterpillars (UNPAR) or parasitized by *C. glomerata* (CG), *C*. *rubecula* (CR) or *Hyposoter ebeninus* (HE). Dark green bars indicate plant treatments with *P. brassicae* caterpillars; light green bars indicate treatments with *P. rapae* caterpillars, white bars indicate undamaged plants. Letters indicate significant differences between treatment groups (GLM, *P* < 0.05)

Parasitism status of the herbivores that induced the plants strongly affected the proportion of hyperparasitized cocoons both at the plant level (Table 2) and at the cocoon clutch level (Online Resource 3, ESM). In contrast, the identity of the *Pieris* caterpillar feeding on the plant did not have a statistically significant effect. Cocoon clutches that were attached to plants previously induced by *C. glomerata–*parasitized *Pieris* caterpillars (CG) were more frequently hyperparasitized than cocoon clutches attached to plants previously damaged by *Pieris* caterpillars carrying *C. rubecula* (CR) or *H. ebeninus* (HE) larvae (Fig. 3). No significant differences between hyperparasitism rates were found for plants previously damaged by *C. rubecula*-parasitized caterpillars or *H. ebeninus*-parasitized caterpillars.

**Table 2:** The effect of herbivore (*Pieris brassicae*, *P. rapae*) and parasitism (*Cotesia glomerata, C. rubecula, Hyposoter ebeninus*, none) on the overall hyperparasitism rates achieved on *C. glomerata* cocoons at the plant level

Model factor	Deviance	Degrees of freedom	*P* value
Overall	431.42	399	
Factor			
Herbivore species (1)	0.056	1	0.8122
Parasitism (2)	19.01	3	< 0.001
Replicate (3)	43.471	4	< 0.001
(1) × (2)	0.899	3	0.8257
(1) × (3)	0.543	4	0.9692
(2) × (3)	5.355	12	0.9450
(1) × (2) × (3)	9.243	12	0.6820

Hyperparasitism was modeled as a binomial occurrence of hyperparasitoids (presence = 1, absence = 0) emerging from the five cocoon clutches attached per plant

Similar results were found when hyperparasitism rates, calculated either at the plant level or at the cocoon clutch level, were restricted only to *L. nana* (Online Resource 4–8, ESM).

## Discussion

To maximize fitness, consumers need to efficiently find suitable resources which in natural environments are often embedded among non-resources. Although foraging strategies of consumers have received considerable attention in plant-based food webs, it is still poorly understood how top carnivores beyond the third trophic level exploit chemical information to locate their resources (Dicke 2009; Poelman and Kos 2016; Aartsma et al. 2019). We found that hyperparasitoids in the fourth trophic level rely on HIPVs to orient themselves in environments containing hosts of different quality as well as non-host parasitoid species which can develop in different herbivore species.

In this study, we investigated a possible multi-parasitoid species scenario that the hyperparasitoid *L. nana* may experience in brassicaecous fields in The Netherlands. When foraging for hosts, *L. nana* may encounter *Pieris* caterpillars parasitized by the gregarious host *C. glomerata*, the solitary host *C. rubecula* or the solitary non-host *H. ebeninus* (Geervliet et al. 2000; Poelman et al. 2014). In laboratory olfactometer bioassays, *L. nana* females preferred HIPVs induced by *C. glomerata*-parasitized caterpillars over those emitted by caterpillars parasitized by *H. ebeninus,* whereas no plant-mediated discrimination occurred between *C. rubecula* and *H. ebeninus*. Thus, *L. nana* females appear to be partially capable of discriminating between hosts and non-hosts based on plant volatile blends released in response to feeding by parasitized caterpillars. The amount of feeding damage inflicted on plants (and thus the quantity of HIPVs released) depends on the parasitism status as well as by parasitoid identity of the attacking caterpillars (Poelman et al. 2011; Cusumano et al. 2018). Yet, bioassays using mechanically treated plants to standardize the amount of damage across treatments have shown the key role played by caterpillar oral secretions on foraging behavior of *L. nana* (Poelman et al. 2012). Thus, hyperparasitoid responses towards HIPVs observed in our study are likely mediated by changes induced by parasitism on composition of caterpillar oral secretions rather than quantitative effects due to differential feeding damage. Chemical analyses of HIPVs induced by parasitized and unparasitized caterpillars in previous studies identified that indeed the composition of HIPVs differs for the parasitism status of the caterpillars and includes variation induced by the identity of the parasitoid species developing inside the caterpillar (Poelman et al. 2012). Future studies should be carried out to identify how non-host parasitoids induce changes in HIPVs to provide a better understanding on the mechanisms behind *L. nana* foraging behavior. The preference for HIPVs induced by caterpillars carrying *C. glomerata* larvae may suggest that host gregarious development is an important trait affecting hyperparasitoid foraging behavior in environments where hosts of different quality co-occur with non-hosts. As hyperparasitoids often face many constraints during the host location process, finding enough resources to sustain the next generation can be challenging: in these situations, adopting a foraging strategy that is finely tuned to target hosts that maximize maternal fitness investments can be adaptive. In the field, complete exploitation of *C. glomerata* broods by *L. nana* females is common and the egg load of *L. nana* closely matches the brood size of C. *glomerata* (15–40 parasitoid larvae/caterpillar) indicating that hyperparasitoid egg load may have evolved to exploit gregarious hosts such as *C. glomerata* (Harvey 2005; Poelman et al. 2012). However, because the host *C. glomerata* and the non-host *H. ebeninus* differ in many traits including developmental lifestyles, how host gregariousness affects *L. nana* discrimination between parasitoid hosts and non-hosts needs to be further investigated. Unfortunately, in the *Brassica*-based food web where this study has been carried out, we were not able to find a hymenopteran parasitoid species which is a non-host for *L. nana* and develops gregariously in *Pieris* caterpillars.

*Lysibia nana* females did not discriminate between the solitary host *C. rubecula* and solitary non-host *H. ebeninus* based on HIPVs induced by parasitized caterpillars. While no other study has yet focused on plant-mediated discrimination between hosts and non-hosts in hyperparasitoids, similar findings have been found for insect parasitoids at the third trophic level. For example, de Rijk et al. (2013) reviewed 26 studies on this topic and found that in 50% of the studies, parasitoids did not discriminate between plants infested with host herbivores or non-host herbivores; thus, non-hosts have potential to reduce foraging efficiency for both parasitoids at the third trophic level and hyperparasitoids at the fourth trophic level. Nonetheless, the similar responses displayed by *L. nana* towards HIPVs emitted by *C. rubecula*-parasitized caterpillars and *H. ebeninus*-parasitized caterpillars may be due to different and non-mutually exclusive, ecological effects. First, olfactometer bioassays and field hyperparasitism rates both hint that *C. rubecula* may be inconspicuous to *L. nana*. In our laboratory bioassays, hyperparasitoid responses to HIPVs emitted by *C. rubecula*-parasitized caterpillars are similar to unparasitized caterpillars; furthermore, overall hyperparasitism rates as well as mortality inflicted by *L. nana* on *C. rubecula* are low compared with *C. glomerata,* at least in cabbage fields located in The Netherlands (Poelman et al. 2012). In addition, it is also possible that *L. nana* can discriminate between *C. rubecula*-parasitized caterpillars and *H. ebeninus*-parasitized caterpillars after landing on the plant. Hyperparasitoids may use body odors of parasitized caterpillars as well as waste products associated with parasitized herbivores (such as honeydew of parasitized aphids) for host localization at the short-range distance (Buitenhuis et al. 2004, 2005; Zhu et al. 2014).

In the field, the pattern of hyperparasitism was similar at the plant and cocoon clutch levels as the highest hyperparasitism rates were consistently found on plants previously induced by *C. glomerata*-parasitized caterpillars. Since plant and cocoon results show a very similar picture, they likely indicate an effect in terms of hyperparasitoid attraction to plant odors, rather than parasitoid arrestment with more frequent ovipositions.

Interestingly, the *Pieris* species in which the different parasitoid species were developing neither affected hyperparasitoid responses to HIPVs, nor field hyperparasitism rates (i.e., cocoon visits, plant visits). It is known that koinobiont endoparasitoids (such as *C. glomerata, C. rubecula* and *H. ebeninus*) affect physiology and metabolism of their herbivore hosts in ways that benefit the parasitoid offspring (Pennacchio and Strand 2006). Evidence is accumulating that host regulation not only affects the caterpillar host but also extends to the herbivore food plant via alterations of the oral secretions of parasitized caterpillars (Poelman et al. 2011; Kaplan et al. 2016; Shikano et al. 2017; Mason et al. 2018). As a conclusion, parasitism can override herbivore identity in terms of plant defense responses and HIPV emission. Indeed, Zhu et al. (2015) showed that *L. nana* females locate *C. glomerata* larvae equally well when developing in *P. brassicae* and in *P. rapae* caterpillars. Our study extends these findings to other host and non-host species that may be encountered under natural conditions by *L. nana*, suggesting that different parasitoid species may rely on similar mechanisms to override herbivore identity at the plant–insect interface. Remarkably, the parasitoid species used in this study do not only inject eggs into their caterpillar hosts but also species-specific polydnaviruses (CgBV, CrBV, or HeIV) which, in addition to regulating host growth, also suppress the immune responses of the herbivores allowing parasitoid offspring to develop (Strand and Burke 2013; Doremus et al. 2014; Drezen et al. 2014). Recent findings have shown that polydnaviruses and not the parasitoid larvae developing within the herbivore body are the major drivers of specific changes induced by parasitism in plant responses including HIPV emission (Cusumano et al. 2018; Tan et al. 2018; Zhu et al. 2018). Future studies should test the hypothesis that the polydnaviruses associated with the parasitoid species studied here are indeed major hidden players that allow *L. nana* to exploit HIPVs in a multi-parasitoid multi-herbivore scenario.

The preferences displayed by *L. nana* in olfactometer bioassays matched hyperparasitism rates (cocoon visits and plant visits) found in common garden experiments. On the one hand, this finding suggests that hyperparasitoid foraging responses obtained in laboratory settings are ecologically meaningful in more complex conditions which better approximate the natural environments where multiple trophic level interactions obviously evolved. On the other hand, this finding hints at a possible direct link between HIPV response and host exploitation efficiency in hyperparasitoid species. The differences in behavioral responses displayed by *L. nana* in the olfactometer towards plant volatiles emitted by *C. glomerata*-parasitized caterpillars and *C. rubecula*-parasitized caterpillars may explain why, in The Netherlands at least, *L. nana* is the main hyperparasitoid species attacking pupae of *C. glomerata* while it is much less frequently associated with *C. rubecula* (Poelman et al. 2012). Nonetheless, *L. nana* offspring perform better on *C. rubecula* than on *C. glomerata* as the higher pupal mass of the solitary host provides more resources for the hyperparasitoid development suggesting a trade-off between cumulative maternal fitness and *per capita* offspring fitness. Yet, the main hyperparasitoids recorded on *C. rubecula* are primary species that attack parasitoid larvae such as *Mesochorus gemellus* and *Baryscapus galactopus* whereas secondary species that oviposit in parasitoid pupae such as *L. nana* are less common (Poelman et al. 2012); this finding could be related to the fact that primary species attack hosts earlier than secondary species and this headstart in resource exploitation is known to often confer a competitive advantage (Harvey et al. 2013; Cusumano et al. 2016). However, whether HIPVs play a role in interspecific competition and resource partition among hyperparasitoid species is unknown.

In terrestrial food webs, plant volatiles are an important source of information which help foraging insects to navigate among complex environments to find suitable resources (Stam et al. 2014). Many studies have shown how plant volatiles are exploited by herbivorous and carnivorous insects to locate hosts, avoid unsuitable resources and detect the presence of competitors (Turlings and Wäckers 2004; Fatouros et al. 2005, 2012). Here, we have shown that plant volatiles are also an important source of information that organisms at the fourth trophic level can use to forage in complex environments where hosts of different quality may be present alongside with non-host resources. Thus, our study contributes to a better understanding of the ecological role that plant volatiles play in structuring species interaction towards the end of the trophic webs.

Hyperparasitoid impacts in basic and applied ecology are likely to be underestimated because these top carnivores are understudied. Increasing our knowledge on hyperparasitoids’ foraging behavior, including the cues they use when foraging in complex environments, is crucial to understand the conditions under which hyperparasitoids can disrupt top-down regulation leading to herbivore outbreaks (Nenzén et al. 2018) and failure of biological pest control programs (Tougeron and Tena 2019).

## Electronic supplementary material

Below is the link to the electronic supplementary material.

Supplementary material 1 (PDF 790 kb)
