# Supplementary material for: Hyperparasitoids exploit herbivore-induced plant volatiles during host location to assess host quality and non-host identity
Source: Oecologia. 2019 Feb 5;189(3):699–709. doi: 10.1007/s00442-019-04352-w (PMC6418317; doi:10.1007/s00442-019-04352-w)
Supplement: Supplementary file 1 — Supplementary material 1 (PDF 790 kb) [file 442_2019_4352_MOESM1_ESM.pdf]

ELECTRONIC SUPPLEMENTARY MATERIAL

To be submitted to: Oecologia

Hyperparasitoids exploit herbivore-induced plant volatiles during host location to assess host quality and non-host identity

Antonino Cusumano[1*], Jeffrey A. Harvey[2,3], Marcel Dicke[1], Erik H. Poelman[1]

[1] Laboratory of Entomology, Wageningen University, P.O. Box 16, 6700 AA, Wageningen, The Netherlands. [2] Department of Terrestrial Ecology, Netherlands Institute of Ecology (NIOO-KNAW), Droevendaalsesteeg 1, 6708 PB, Wageningen, The Netherlands, [3] VU University Amsterdam, Department of Ecological Sciences, Section Animal Ecology, De Boelelaan 1085, 1081 HV Amsterdam, The Netherlands

*correspondence to: Antonino Cusumano, Department of Plant Sciences, Laboratory of Entomology, Droevendaalsesteeg 1, 6708PB, WAGENINGEN, Phone: +31 317 484 148, Email: cusumanoantonino@tiscali.it

Online Resource 1. Proportion of each species of hyperparasitoid collected in the common garden experiments from *Cotesia glomerata* cocoon clutches attached to *Brassica oleracea* “Kimmeridge” plants subjected to different induction treatments. Colors indicate different hyperparasitoid species: *Lysibia nana* (blue); *Acrolyta nens* (red); *Gelis agilis* (green); *Gelis* sp. (purple); *Pteromalus* sp. (light blue).

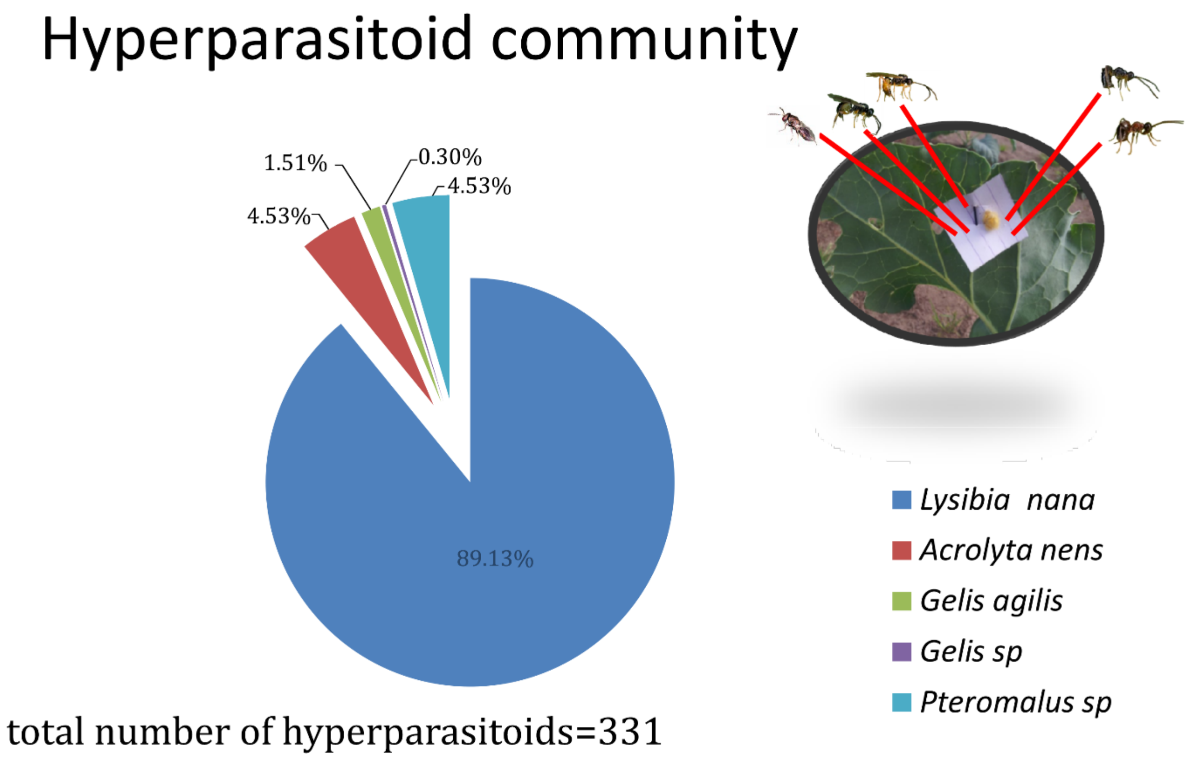

Online Resource 2. The effect of plant induction treatment on the overall hyperparasitism rates achieved on *Cotesia glomerata* cocoons at the clutch level. Hyperparasitism was modeled as the number of clutches yielding hyperparasitoids out of the fixed totals of five cocoon clutches attached to the plant (see main text for information about statistical analyses).

**Model factor**	**Deviance**	**Degrees of freedom**	***P* value**
**Overall (cocoon level)**	427.74	499	
**Factor**			
**Induction treatment (1)**	43.604	8	<0.001
**Replicate (2)**	55.847	4	<0.001
**(1) × (2)**	14.251	32	0.9971

Online Resource 3. The effect of the herbivore species (*Pieris brassicae*, *P. rapae*) and parasitism (*Cotesia glomerata, C. rubecula, Hyposoter ebeninus,* none) on the overall hyperparasitism rates achieved on *C. glomerata* cocoons at the clutch level. Hyperparasitism was modeled as the number of clutches yielding hyperparasitoids out of the fixed totals of five cocoon clutches attached to the plant.

**Model factor**	**Deviance**	**Degrees of freedom**	***P* value**
**Overall (cocoon level)**	366.17	399	
**Factor**			
**Herbivore species (1)**	0.086	1	0.7695
**Parasitism (2)**	21.875	3	<0.001
**Replicate (3)**	50.353	4	<0.001
**(1) × (2)**	2.012	3	0.5700
**(1) × (3)**	0.512	4	0.9724
**(2) × (3)**	4.376	12	0.9757
**(1) × (2) × (3)**	7.326	12	0.8

Online Resource 4. Percentage of *Cotesia glomerata* cocoon clutches that yielded *Lysibia nana* hyperparasitoids in the field trials either at the plant level (i.e. at least one cocoon clutch out of the five clutches attached to the plant contained *L. nana* hyperparasitoids) (left) or at the individual clutch level (right). The cocoons were collected from plants that were either left untreated (UD), infested with unparasitized *Pieris* caterpillars (UNPAR) or parasitized by *C. glomerata* (CG), *C. rubecula* (CR) or *Hyposoter ebeninus* (HE). Dark green bars indicate plant treatments with *P brassicae*; light green bars indicate treatments with *P. rapae*, white bars indicate undamaged plants). Letters indicate significant differences between treatment groups (GLM, P < 0.05).

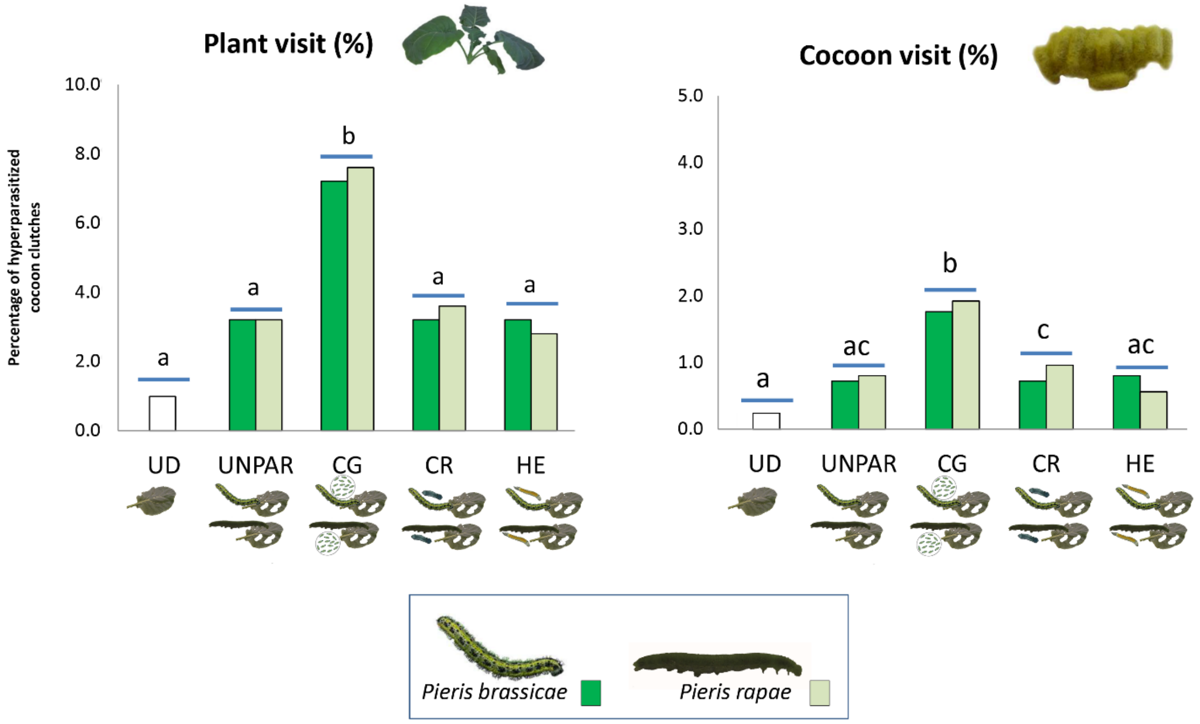

Online Resource 5. The effect of plant induction treatment on the hyperparasitism rates achieved by *Lysibia nana* on *Cotesia glomerata* cocoons at the plant level. Hyperparasitism was modeled as a binomial occurrence of *L. nana* hyperparasitoids (presence=1, absence=0) emerging from the five cocoon clutches attached per plant

**Model factor**	**Deviance**	**Degrees of freedom**	***P* value**
**Overall (plant level)**	474.41	499	
**Factor**			
**Induction treatment**	36.577	8	<0.001
**Replicate**	46.770	4	<0.001
**(1) × (2)**	16.995	32	0.9865

Online Resource 6 The effect of plant induction treatment on the hyperparasitism rates achieved by *Lysibia nana* on *Cotesia glomerata* cocoons at the clutch level. Hyperparasitism was modeled as the number of clutches yielding *L. nana* hyperparasitoids out of the fixed totals of five cocoon clutches attached to the plant (see main text for information about statistical analyses).

**Model factor**	**Deviance**	**Degrees of freedom**	***P* value**
**Overall (cocoon level)**	405.21	499	
**Factor**			
**Induction treatment**	40.061	8	<0.001
**Replicate**	52.702	4	<0.001
**(1) × (2)**	14.670	32	0.9963

Online Resource 7. The effect of herbivore (*Pieris brassicae*, *P. rapae*) and parasitism (*Cotesia glomerata, C. rubecula, Hyposoter ebeninus*, none) on the hyperparasitism rates achieved by *Lysibia nana* on *C. glomerata* cocoons at the plant level. Hyperparasitism was modeled as a binomial occurrence of *L. nana* hyperparasitoids (presence=1, absence=0) emerging from the five cocoon clutches attached per plant

**Model factor**	**Deviance**	**Degrees of freedom**	***P* value**
**Overall (plant level)**	416.41	399	
**Factor**			
**Herbivore species (1)**	0.059	1	0.8077
**Parasitism (2)**	17.862	3	<0.001
**Replicate (3)**	42.737	4	<0.001
**(1) × (2)**	0.359	3	0.9486
**(1) × (3)**	3.112	4	0.5393
**(2) × (3)**	6.005	12	0.9158
**(1) × (2) × (3)**	6.088	12	0.9116

Online Resource 8. The effect of the herbivore species (*Pieris brassicae*, *P. rapae*) and parasitism (*Cotesia glomerata, C. rubecula, Hyposoter ebeninus,* none) on the hyperparasitism rates achieved by *Lysibia nana* on *C. glomerata* cocoons at the clutch level. Hyperparasitism was modeled as the number of clutches yielding *L. nana* hyperparasitoids out of the fixed totals of five cocoon clutches attached to the plant.

**Model factor**	**Deviance**	**Degrees of freedom**	***P* value**
**Overall (cocoon level)**	347.83	399	
**Factor**			
**Herbivore species (1)**	0.165	1	0.6842
**Parasitism (2)**	19.407	3	<0.001
**Replicate (3)**	48.158	4	<0.001
**(1) × (2)**	1.392	3	0.7073
**(1) × (3)**	3.208	4	0.5236
**(2) × (3)**	5.383	12	0.9439
**(1) × (2) × (3)**	4.420	12	0.97
